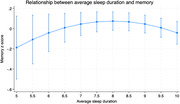# Sleep and Neurocognitive Performance in Middle‐Aged Latina Women in an Agricultural Community: Insights from the CHAMACOS Maternal Cognition Study

**DOI:** 10.1002/alz.093162

**Published:** 2025-01-09

**Authors:** Nadia Rojas, Lucia Calderon, Katherine Kogut, Maria Teresa Rodriguez, Monica Romero, Lizari Garcia, Hector Rodriguez, Jenny Guadamuz, Brenda Eskenazi, Hector M González, Jacqueline M Torres

**Affiliations:** ^1^ University of California, Berkeley, Berkeley, CA USA; ^2^ University of California, San Francisco, San Francisco, CA USA; ^3^ La Clínica de Salud del Valle de Salinas, Salinas, CA USA; ^4^ UC Berkeley School of Public Health, Berkeley, CA USA; ^5^ University of California, San Diego, La Jolla, CA USA; ^6^ University of California San Francisco, San Francisco, CA USA

## Abstract

**Background:**

Latino adults face a 1.5‐to‐2‐fold increased risk of ADRDs compared to non‐Hispanic whites. Sleep disturbances among Latinos have been linked with worse neurocognitive function and a higher risk for ADRD. Limited research exists on sleep and neurocognitive function among middle‐aged Latinas. This study addresses these gaps by exploring the relationship between sleep duration, quality, nap frequency, and cognitive function in Latinas living in an agricultural community in California.

**Method:**

Data come from the Center for the Health Assessment of Mothers and Children of Salinas (CHAMACOS) Maternal Cognition study, which included 513 middle‐aged Latina women (mean age: 48.8) living in an underserved agricultural community in California. Average nightly sleep duration, sleep quality, and nap frequency were measured via self‐report. Participants completed the SOL‐INCA Neurocognitive Assessment, which yielded global and domain‐specific cognitive z‐scores (memory, executive function, verbal fluency). We separately estimated associations between each sleep measure and cognitive scores, controlling for socio‐demographic and health‐related covariates. In models of continuous hours of nightly sleep, we considered non‐linear associations.

**Result:**

Participants reported a mean of 8.6 hours of nightly sleep; nap frequency was 64.8% no naps, 22.2% 1‐2 naps, 7.2% 3‐4 naps, and 5.9% 5+ naps. Sleep quality was 48.5% very sound or sound, 30.10% average, and 21.36% restless or very restless. We observed an inverted U‐shaped association between sleep duration and memory, executive function, and global cognitive z‐scores. Taking 1 or 2 naps per week (vs. zero naps) was associated with higher memory z‐scores (Coef: 0.21, 95% CI: 0.02, 0.39), while napping 5 or more times per week was associated with lower memory z‐scores (Coef: ‐0.61, 95% CI: ‐0.94, ‐0.29) and global cognitive function z‐scores (Coef: ‐0.25, 95% CI: ‐0.46, ‐0.04). We did not observe associations between self‐reported sleep quality and neurocognitive scores.

**Conclusion:**

Our findings emphasize the significant connection between multiple measures of sleep duration, quality, and neurocognitive function. The results from this study can provide valuable insights for developing interventions targeted at improving sleep among Latinas living in rural settings. Interventions that target improved sleep may benefit neurocognitive function among middle‐aged Latinas living in agricultural settings.